# Medical care at a mass gathering music festival: Retrospective study over 7 years (2011–2017)

**DOI:** 10.1007/s00508-021-01856-5

**Published:** 2021-04-26

**Authors:** Mathias Maleczek, Simon Rubi, Christian Fohringer, Georg Scheriau, Elias Meyer, Thomas Uray, Andreas Duma

**Affiliations:** 1 Department of Anaesthesiology, Intensive Care Medicine and Pain Medicine, Medical University of Vienna, Waehringer Guertel 18–20/9i, 1090 Vienna, Austria; 2 NOTRUF NOE GmbH, St. Poelten, Austria; 3 Section for Medical Statistics, Center for Medical Statistics, Informatics, and Intelligent Systems (CeMSIIS), Medical University of Vienna, Vienna, Austria; 4 Department of Emergency Medicine, Medical University of Vienna, Vienna, Austria

**Keywords:** Mass casualty incidents, Disaster planning, Emergency medical services, Triage, Insect bites and stings

## Abstract

**Background:**

Knowledge about longitudinal changes in epidemiological data at mass gathering events is sparse. The goal of this study was to determine and compare the type, severity and frequency of illnesses at a large music festival over 7 consecutive years (2011–2017).

**Methods:**

Prospectively collected data from the rescue operation protocols of an Austrian music festival were retrieved and analyzed. Patient presentation rates (PPR) and transport to hospital rates (TTHR) were calculated and compared between years. Linear regression was used to investigate the association between (a) total number of visitors and number of patient presentations, and (b) environmental factors and temperature related medical emergencies. A descriptive analysis of pertinent medical logistics management was performed.

**Results:**

The median (minimum to maximum) PPR and TTHR were 12.01 (9.33 in 2016 to 20.86 in 2011) and 0.57 (0.40 in 2017 to 1.06 in 2013) per 1000 visitors, respectively. In linear regression models, no significant associations were found between the number of visitors and either the total number of patient presentations, NACA 1–2 or NACA 3–5 classified emergencies.

Environmental temperature had a significant impact on heat related patient presentations (*p* < 0.001).

**Conclusion:**

There were significant differences and a high variance in both PPR and TTHR over the years. Contrary to our expectations, the number of visitors did not predict the number of patient presentations. Ambient temperature was associated with the number of heat related emergencies but not with the number of cold related emergencies. Prevention strategies, such as the removal of insect nests, resulted in significantly fewer insect related emergencies.

## Introduction

Mass gathering music festivals are increasing in popularity. In 2019, 26% of adults in the UK visited a music festival [1]. Projected medical emergency case numbers as well as an ever-present potential for mass causalities during such music festivals, necessitate the presence of well-organized, professional, on-site medical services [2]. Achieving valid estimations of patient presentation rates (PPR) and medical emergencies is necessary for effective logistical planning; however, incidence rates are associated with both event-dependent factors, such as music genre and visitor demographics, and environment-dependent factors, such as weather and landscape [3, 4]. Reported PPRs vary extensively among mass gathering events, from 0.7 per 1000 visitors to 72 per 1000 visitors [5, 6]. Prediction of the incidence of medical interventions across music festivals is, therefore, inaccurate; however, it remains unclear if and to what extent, PPR, TTHR and epidemiology change year by year at the same festival.

Most studies reported patient presentations at a given music festival for only 1 or 2 years [6–10], and/or focus on special topics, such as the mental health of visitors or drug use [6, 7, 11, 12]. Longitudinal observations from music festivals over several consecutive years are sparse [10]. It is unclear (yet important for predicting medical demand) if the epidemiology of health related events at an annual music festival changes over the course of several years. The main aim of this study was to determine longitudinal changes in PPR, TTHR and pertinent epidemiology over a 7-year span at a large pop and electronic music festival in Austria which draws up to 200,000 visitors per annum. Since event-dependent factors do not tend to undergo major changes from year to year, potential longitudinal changes in PPR and TTHR may possibly be associated with environment-dependent factors. Therefore, the influence of weather on epidemiology was explored.

## Patients, materials and methods

### Design and setting

A retrospective study of prospectively acquired patient data from the Frequency Festival was conducted, from the years 2011–2017. This annual outdoor music festival takes place every August, over a course of 3–4 days in the same area of St. Poelten, a city in north east Austria. Between 115,000 and 200,000 persons attend the festival each year. For the years pertinent to this study, on-site medical services were provided by the Austrian Red Cross (ARC). The ARC was on-site for 4–5 days every year, including the day of the festival’s start and/or finish. Data were recorded using the local medical dispatching agency’s *Notruf Niederoesterreich* software (St. Poelten, Austria).

The study was approved by the Ethics Committee of the Medical University of Vienna (1496/2016) and registered at clinicaltrials.org (NCT03570151). Need for informed consent was waived as this was a retrospective study.

### Organization and operational management of the medical emergency service

**Fig. 1:**
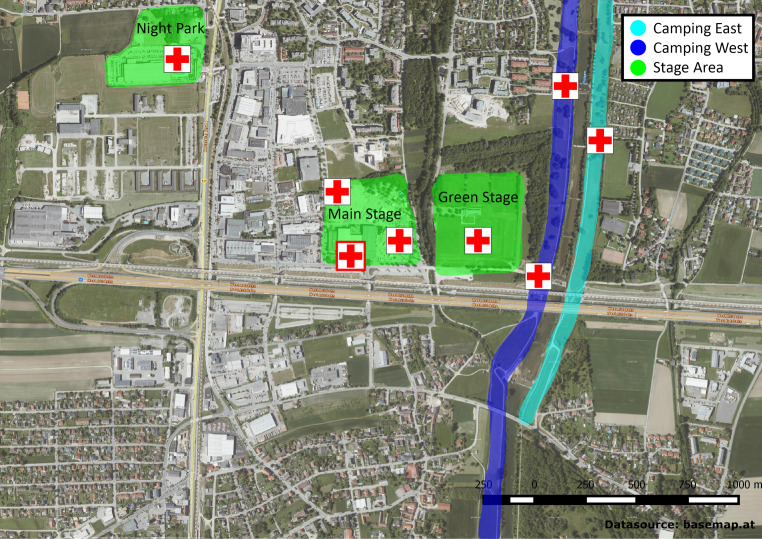
Map of the Frequency Festival. Camp sites (*blue*, *turquoise*) account for 30 ha along a small river. From there, the main stage area can be reached by foot in approx. 15 min. This area was visited by day guests, as well as campers. *Red crosses* symbolize medical aid points. The main first aid post is symbolized by a *red cross surrounded by red rectangle*. Datasource: basemap.at

As mentioned above, on-site emergency medical care was provided by the ARC. The festival area itself consisted of an event area and a camping area (Fig. 1), with eight first aid posts, each staffed with up to four emergency medical technicians (EMT). There was one main first aid post, consisting of two resuscitation pods and eight intermediate care pods equipped to monitor patients and perform advanced cardiac and trauma life support, comparable to a level 1 hospital. Adjacent to this main post and housed in a standard ship container was the mobile command center. Patients in need of emergency physician (EP) consultation were either moved to the main first aid post or an EP was driven directly to the site of injury.

To minimize heat exhaustion, free drinking water was provided on site by the festival organizer.

All dispatchments were recorded electronically.

At the beginning of the Frequency Festival a combination of Maurer’s formula and experiences from other music festivals were used for planning of emergency medical services (EMS) capacity. In the subsequent years, the capacity planning was primarily based on experience from preceding years.

### Study population

The study population consisted of all on-site patients. Medical information including patient history and transport route about those seeking medical assistance was obtained. All other persons at the festival site were defined as not needing medical help.

All points of patient contact, as well as all patient transfers, both between medical aid posts and to hospitals, were recorded.

Patient transfers with incomplete data sets were excluded.

### Measurements

Sex, age, chief complaint, reason for complaint, the body region involved, the location on the festival grounds where the injury happened, the first aid post or hospital the patient was brought to, and the time(s) and date(s) of patient contact were extracted from the raw data. The EMT case protocol documentation was also included. One of the authors, Christian Fohringer, was interviewed as head physician on site during the study period in order to obtain additional information on possible medical presentation accumulation patterns, their management and possible organizational pitfalls.

The data were processed by three members of the study team. Medical emergencies were categorized by chief complaint, reason for complaint and involved body region (Table 1).

**Table 1:** Type of emergencies. The categories used to judge emergencies are shown. Each patient was categorized by chief complaint, the cause of complaint and body region. If applicable, multiple items from all 3 categories were extracted

Chief complaint	Reason for complaint	Body region
Altered mental state	Allergy	Ear, nose and throat
Atraumatic bleeding	Burn (1°, 2°, 3°)	Face
Diarrhea/vomiting	Circulation (transient loss of consciousness, TLOC)	Gastrointestinal tract
Dizziness	Hyperthermia	Gynecological
Dyspnea	Hypothermia	Head/neck
Exanthema	Infection	Heart/chest
Fever	Insect bite	Lower extremity
Pain	Intoxication (alcohol, other)	Skin
Psychiatric	Metabolic	Teeth
Unknown	Neurological emergency	Torso
	Pain of unknown origin (not meeting any other criteria like trauma, …)	Upper extremity
	Seizure	Urological
	Traumatic (blunt, penetrating, unknown)	
	Upper respiratory tract Infections	

Emergencies were graded using a modified National Advisory Committee on Aeronautics (NACA) score [13]. This score consists of seven numbered categories (representing the severity of a given medical emergency) ranging from minor disturbance (NACA-1) to death (NACA-7). An eighth category was introduced to grade patients receiving follow-up treatment. Medical management was categorized into (a) no treatment, (b) treatment at site, (c) transfer to hospital and (d) follow-up at site.

Weather data were acquired from the Austrian national weather service (*Zentralanstalt fuer Meteorologie*, ZAMG, Vienna). The data set received consisted of local temperature (at 07:00, 14:00 and 19:00 h), the minimum, maximum and mean local temperatures and the daily precipitation in millimeters.

### Study endpoints

The primary endpoints were PPR (the number of individuals seeking help per 1000 visitors) and TTHR (the number of hospitalizations per 1000 visitors). For this study, all persons at the festival site were counted as visitors; this includes organizers and performers.

The secondary endpoints were the NACA scores, stratified into: NACA 1–2 (ambulatory patients), NACA 3–5 (patients in need of advanced care including resuscitation) and NACA 6–7 (patients in acute need of cardiopulmonary resuscitation). Tallies of the various NACA score categories and incidence rates for the respective groups were calculated. Possible correlations that weather and visitor number have with medical emergencies was investigated. Incidence rates and counts of chief complaints (including traumatic and drug related presentations) were calculated. The number of insect related emergencies was compared over the years in order to determine the effect of on-site insect prevention. The organizational and operational management of the on-site medical emergency services were described.

### Statistics

The epidemiological and demographic data of presenting patients were collected. The PPR and TTHR were calculated both per year and as an overall value per 1000 visitors. Changes in PPR and TTHR per year were investigated using χ^2^-tests; the medians and ranges (minimum to maximum) of PPR and TTHR were also calculated.

Additionally, we wanted to investigate the distribution of NACA scores over time. Therefore, a χ^2^-test was calculated. To show the change in total amount of NACA 3–5 emergencies over the years, a linear regression analysis was performed using the year as an independent variable and the incidence of NACA 3–5 emergencies as a dependent variable.

The influence of the number of visitors on the total number of patient presentations was investigated using linear regression, using the total number of visitors as an independent variable and the number of emergencies, the amount of NACA 1–2 and the amount of NACA 3–5 emergencies as dependent variables.

Furthermore, the possible influence that average temperatures and yearly precipitation may have on the amount of temperature related medical emergencies was of interest. A linear regression analysis was computed using average temperatures and average precipitation as independent variables, and the percentage of temperature related emergencies as a dependent variable.

Descriptive statistics were performed to show the distribution of chief complaints, and to further detail traumatic and drug related causes for patient presentation.

Due to a reported accumulation of wasp nests in 2011 and their preventive removal in the following years, the effect of this action on insect related emergencies was analyzed using a χ^2^-test.

Two linear regressions were computed, using average temperature and average precipitation per year as independent variables and the percentage of cold related and heat related emergencies as dependent variables.

All statistical calculations were made using R (R: A Language and Environment for Statistical Computing, Vienna, 2018).

## Results

A total of 1,003,500 persons visited the festival between 2011 and 2017. Of 18,684 medical records 13,759 were included. Due to either incomplete data (2605 presentations, 14%) or multiple entries (2320 presentations, 12%) 4925 cases had to be excluded. The cause for these multiple entries was the method of documentation: Every time a patient was moved to a new location (e.g., from outpost to main post), a new entry was made by the dispatch system. As such, double entries were merged into one entry consisting of all available information.

The median (minimum to maximum) PPR and TTHR were 12.01 (9.33–20.86) per 1000 visitors and 0.57 (0.40–1.06) per 1000 visitors, respectively (Table 2). The PPR and TTHR changed significantly over the years, although demographics of the festival population, such as sex and age, did not change by year. Table 3 shows the frequencies of chief complaints. Most of the presentations (65%) were due to pain (of any origin). The most common reason for pain was of traumatic origin (70%) (Table 4). Trauma was the number 1 reason for presentations, accounting for 6466 presentations (47%). Most of those trauma patients had pain as their chief complaint. Most traumatic presentations over the years were caused by blunt trauma (57%), followed by penetrating trauma (20%). Most penetrating trauma was caused by pieces of metal simply lying on the ground. No gunshot wounds were recorded. The most common causes of blunt trauma were falls and blisters on feet and hands. A small fraction of blunt trauma due to fights was recorded. The majority of both blunt and penetrating trauma was categorized as either NACA 1 or 2 and was treated on-site. In 20.9% of the traumatic presentations, the cause was not documented.

**Table 2:** Distribution of patient presentation by year. Median patient presentation rate and transport to hospital rate, as well as patient demographic data by year

Year	Length (days)	Visitors (*n*)	Patient presentations	PPR	Male (*n*)	Female (*n*)	Age (years, mean)	Transported patients (*n*)	TTHR
2011	4	137,500	2868	20.86	1469	1091	20	105	0.76
2012	5	160,000	2091	13.07	1124	947	21	74	0.46
2013	5	135,000	2659	19.70	1571	1087	21	143	1.06
2014	5	200,000	2015	10.08	1139	845	21	114	0.57
2015	5	115,000	1374	11.95	729	628	20	68	0.59
2016	5	120,000	1119	9.33	613	495	22	53	0.44
2017	4	136,000	1633	12.01	807	819	21	55	0.40
*Sum*		*1,003,500*	*13,759*	$\overline{x}$ *=* *12.01*	*7452*	*5912*	*21*	*612*	$\overline{x}$ *=* *0.57*

*n* number, *PPR* patient presentation rate, *TTHR* transport to hospital rate, $\overline{x}$ median

**Table 3:** Epidemiological data of health related events at the festival. Absolute and relative frequency of the chief complaint, having more than one chief complaint resulted in double entries

Year		2011	2012	2013	2014	2015	2016	2017	SUM
*Presentations total*	*n*	2868	2091	2659	2015	1374	1119	1633	*13,759*
*Pain*	*n*	1902	1299	1637	1197	941	792	1142	*8910*
	%	66%	62%	62%	59%	69%	71%	70%	*65%*
*Altered mental state*	*n*	129	106	189	204	135	76	69	*908*
	%	5%	5%	7%	10%	10%	7%	4%	*7%*
*Dizziness*	*n*	129	84	119	74	63	29	115	*613*
	%	5%	4%	5%	4%	5%	3%	7%	*5%*
*Atraumatic bleeding*	*n*	10	11	21	12	5	7	7	*73*
	%	<1%	1%	1%	<1%	<1%	1%	<1%	*1%*
*Exanthema*	*n*	33	22	24	25	21	18	35	*178*
	%	1%	1%	1%	1%	2%	2%	2%	*11%*
*Dyspnea*	*n*	17	13	19	18	14	8	11	*100*
	%	1%	1%	1%	1%	1%	1%	1%	*1%*
*Diarrhea*	*n*	88	70	70	53	57	40	65	*443*
	%	3%	3%	3%	3%	4%	4%	4%	*3%*
*Psychiatric*	*n*	12	6	18	14	12	13	21	*96*
	%	<1%	<1%	1%	1%	1%	1%	1%	*1%*
*Fever*	*n*	6	11	13	15	13	7	9	*74*
	%	<1%	1%	1%	1%	1%	1%	1%	*1%*
*Unknown*	*n*	237	180	286	176	15	56	51	*1001*
	%	8%	9%	11%	9%	1%	5%	3%	*7%*
*Follow-up treatment*	*n*	307	294	263	229	100	81	110	*1384*
	%	11%	14%	10%	11%	7%	7%	7%	*10%*

*n* number

**Table 4:** Reasons for pain. As pain was the most common reason for presentation, with 8910 patients, the following table shows the 5 most common reasons for pain in the presenting patients over all 7 years

*n*	%	Reason of complaint
6241	70	Trauma, overall
3587	40	Trauma, blunt
1289	14	Trauma, unknown
1264	14	Trauma, penetrating
734	8	Insect bites
728	8	Pain of unknown origin
269	3	Ear, nose and throat
214	2	Burns, 1°

*n* number

Only 5% of presentations (746 over 7 years) were due to intoxication, with the majority of these (86%) being due to alcohol. The remaining 14% (104 cases) were due to recreational drugs.

**Fig. 2:**
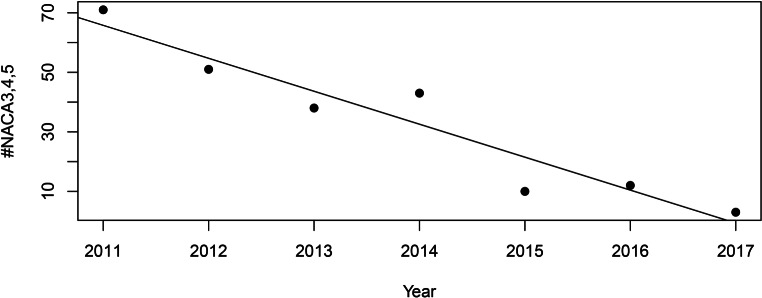
NACA 3–5 emergencies by year. This dot plot shows the number of NACA 3–5 emergencies by year. There was a linear decline during the study period. (#: number)

**Fig. 3:**
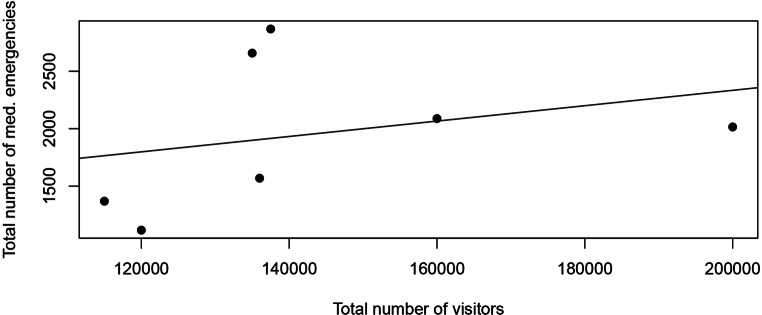
Total number of medical emergencies with respect to the total number of visitors. This dot plot shows the association of total number of emergencies and the number of visitors. No correlation could be found leading to the conclusion that the number of emergencies is not dependent on the number of visitors in this sample over 7 years

The NACA scores were not equally distributed over time (*p* < 0.01). There were no NACA 6–7 cases documented during the study period. Although the number of visitors did not decrease, the absolute number of NACA 3–5 emergencies decreased by 95%, from 73 to 3, over the years (*p* < 0.001) (Fig. 2). After linear regression analysis, the total number of visitors was not observed to have any significant effect (*p* = 0.52) on the number of medical emergencies, even when stratified for NACA 1–2 and NACA 3–5 cases (Fig. 3).

Regarding the organization of on-site emergency medical care, the main first aid pod was staffed with up to 3 EPs and up to 8 EMTs. During concert hours, a third EP was at the site. Additionally, 30–55 EMTs were deployed at all times in mobile first aid squads, to patrol the festival area. The minimum number of personnel on site did not differ over the years, although additional volunteer EMTs were on site irregularly.

### Environmental factors

One of the authors, Christian Fohringer, acted as leading emergency physician at the Frequency Festival during the study period, and possessed detailed knowledge of special precautions taken by the organizers. These included preventing insect related emergencies by removing wasp nests. In 2011, 395 insect related emergencies were documented. Beginning in 2012, wasp nests were sought out and removed, resulting in only 44 insect stings that year (*p* < 0.01). In subsequent years, the numbers varied between 41 (2014) and 187 (2013).

Using the abovementioned linear regression model, only the average temperature was found to have a significant impact on the percentage of heat related medical emergencies, with the percentage of emergencies increasing with temperature (*p* < 0.01). The same calculation done with cold related emergencies showed no significant correlation.

## Discussion

Our study is the first to examine the medical emergencies within the visitor population of a large music festival over 7 consecutive years. To our knowledge, only one study has investigated a college music festival over a 4-year period [10].

Our data show that between 9.33 (2016) and 20.86 per 1000 visitors (2011) were in need of emergency medical services, resulting in 0.40 (2017) and 1.06 per 1000 visitors (2013) being transported to hospital. These numbers varied significantly between various years. The PPRs that we observed landed squarely within the PPR ranges described in the literature [5, 6]. Concerning TTHR, reported numbers ranged from 0.71 to 1.8 per 1000 visitors [5, 6].

There is no standardized and evidence-based method of planning the emergency medical care for a music festival. Certain precautions are recommended but the organization is widely done by local emergency medical services [14]. Calculation of the number of personnel or amount of equipment needed relies on local laws, previous experiences and protocols such as the Maurer formula in German speaking countries, which relies mainly on the number of visitors, the location and type of event [15].

Remarkably, there was no significant correlation between the number of visitors and patient presentations. The theory that this is caused by some kind of ceiling effect, occurring when the time spent for queuing up for a small wound dressing becomes too long, was disproven by analyzing NACA I–II and NACA III–V cases separately, following the assumption that a severely sick or injured patient will not have to wait in line. Even in the NACA III–V group, no correlation between visitor numbers and patient presentations was found.

This lack of a correlation raises important questions for future event planning. It may be that the necessary personnel calculated from Maurer’s formula tends to overestimate; this requires revaluation in future studies. When considering what may influence the PPR and TTHR of the Frequency Festival, the weather forecast seems to be more important than the number of visitors. In the year 2011, there were significantly more insect related cases; this included all kinds of bites/stings/etc. In that year, a lot of wasp nests were found in the area, including the camping grounds. In the following years, the organizers had those nests removed before the beginning of the festival. This simple action proved effective. The number of insect related incidents sank significantly. This is one of the precautions which led to a relatively low number of patient presentations. In accordance with the literature [12] we could show that prevention strategies reduce the number of patient presentations in the group in question.

In our study, the reported percentage of alcohol related and drug related emergencies was only 5%; this is a considerably lower number than has been reported elsewhere in the literature for similar events [12, 16]. This may be either founded in the high proportion of basic traumatic emergencies, or the possibility of sleeping off the intoxication at the site.

There were no cardiac arrests seen during the observational period. A cross-check with regional news outlets/publications around the dates of the festival brought no further information about possible deaths occurring at the festival. Although literature analysis showed that mortality is reported to be a factor in festival visitor populations [2, 17, 18], no deaths were reported in this sample. The reported causes of death in the literature include mass casualty events subsequent to trampling, structural collapse and acts of terror. Grange et al. report that all deaths in their sample of over 400 occurred at classical concerts, although the PPR was highest at rock concerts [4]. Other authors have reported deaths due to intoxications at various festivals [8]. In our reported sample, the number of intoxications was quite low, which—in combination with visitors’ relatively young age and the friendly atmosphere—could have contributed to zero deaths being reported.

### Limitations

The biggest limitation and source of bias in our study is the nature of the inquiry into the data itself, as we were forced to trust the information given to us. As such medical documentation is mandatory for all EMTs in Austria by law, we decided to use it despite its possible shortcomings and our information processing yielded consistent data for almost all cases. Where there was clear doubt about the documentation, the patient presentation was either excluded or categorized as it seemed appropriate. These questionable incidents of documentation occurred, among other reasons, due to an occasional tendency of EMTs to overtriage. For example, a patient who was documented as having severe anaphylactic shock but was not transported to a hospital, was downgraded by the authors to having had simply an allergic reaction. Except in severe intoxications, the influence of alcohol or degree of intoxication was not documented in trauma cases.

Even though 26% of database entries were excluded, the risk of exclusion bias is, in our opinion, low. Of the entries 12% were duplicates, and therefore their inclusion would have introduced bias. Incomplete data were the reason for exclusion in 14% of cases, one which remained fairly constant over the years. Those cases were excluded resulting in a justifiable loss of information, especially considering that retrospective retrieval of this data is impossible. Additionally, for most cases exhibiting incomplete data entries visitors were administrated at the medical site by error.

Retrospective detection and differentiation of drug use is difficult to achieve. To differentiate between intoxication due to alcohol, recreational drugs or both, the documented patient history was used. This represents a study limitation, as this assessment is likely to be flawed, many intoxications include a combination of alcohol and illicit substances and some patients will not admit to drug use. This could have contributed to an inaccurately low reported number of intoxicated patients.

A further limitation lies in the fact that no exact visitor numbers, only published estimates, are made available by the festival’s organizer.

## Conclusion

There were significant differences and a high variance in both PPR and TTHR over the years. Contrary to our expectations, the number of visitors did not predict the number of patient presentations. Ambient temperature was associated with the number of heat related emergencies, but not with the number of cold related emergencies. Prevention strategies, such as the removal of insect nests, resulted in significantly fewer insect related emergencies.

## Abbreviations

ARC: Austria Red Cross
EMS: Emergency medical services
EMT: Emergency medical technician
EP: Emergency physician
NACA: National Advisory Committee on Aeronautics (score)
PPR: Patient presentation rate
TTHR: Transfer to hospital rate
